# Observation of Dark States in Two-Dimensional Electronic Spectra of Chlorosomes

**DOI:** 10.1021/acs.jpcb.4c00067

**Published:** 2024-04-03

**Authors:** Vesna Erić, Xinmeng Li, Lolita Dsouza, Annemarie Huijser, Alfred R. Holzwarth, Francesco Buda, G. J. Agur Sevink, Huub J. M. de Groot, Thomas L. C. Jansen

**Affiliations:** † Zernike Institute for Advanced Materials, University of Groningen, 9747 AG Groningen, The Netherlands; ‡ Department of Chemistry and Hylleraas Centre for Quantum Molecular Sciences, University of Oslo, Sem Sælands vei 26, 0315 Oslo, Norway; § Leiden Institute of Chemistry, Leiden University, Einsteinweg 55, 2300 RA Leiden, The Netherlands; ∥ MESA+ Institute for Nanotechnology, University of Twente, Drienerlolaan 5, 7522 NB Enschede, The Netherlands; ⊥ Department of Biophysical Chemistry, Max Planck Institute for Chemical Energy Conversion, Stiftstraße 34-36, 45470 Mülheim, Germany

## Abstract

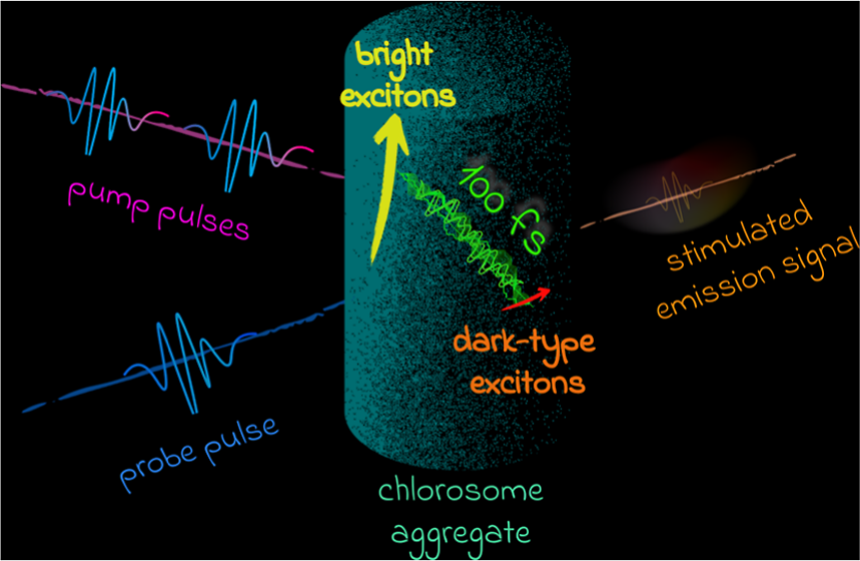

Observations of low-lying dark states in several photosynthetic complexes challenge our understanding of the mechanisms behind their efficient energy transfer processes. Computational models are necessary for providing novel insights into the nature and function of dark states, especially since these are not directly accessible in spectroscopy experiments. Here, we will focus on signatures of dark-type states in chlorosomes, a light-harvesting complex from green sulfur bacteria well-known for uniting a broad absorption band with very efficient energy transfer. In agreement with experiments, our simulations of two-dimensional electronic spectra capture the ultrafast exciton transfer occurring in 100s of femtoseconds within a single chlorosome cylinder. The sub-100 fs process corresponds to relaxation within the single-excitation manifold in a single chlorosome tube, where all initially created populations in the bright exciton states are quickly transferred to dark-type exciton states. Structural inhomogeneities on the local scale cause a redistribution of the oscillator strength, leading to the emergence of these dark-type exciton states, which dominate ultrafast energy transfer. The presence of the dark-type exciton states suppresses energy loss from an isolated chlorosome via fluorescence quenching, as observed experimentally. Our results further question whether relaxation to dark-exciton states is a leading process or merely competes with transfer to the baseplate within the photosynthetic apparatus of green sulfur bacteria.

## Introduction

The process of photosynthesis starts with light capture, followed by the fast transfer of excitation energy through the photosynthetic apparatus toward the reaction center, where the initial charge separation occurs.^1^ Since photosynthetic organisms thrive in environments with different light conditions, nature offers a variety of solutions for efficient light harvesting.^2−4^ Variations in structures of photosynthetic complexes are responsible for successful light capture and energy transfer.^2^ The excitation energy is transferred through a disordered and fluctuating energy landscape in complex molecular aggregates. Still, there are questions about mechanisms that allow these organisms to utilize the fluctuations of the disordered environment to enhance the efficiency of energy transfer. Low-lying dark-type states play a significant role in the efficiency of ultrafast energy transfer in photosynthetic complexes.^5^ The possibility of characterizing and directly studying these states is restricted since they are not directly accessible in spectroscopy experiments. Hence, there is uncertainty about the nature and selection rules that forbid radiative transition to such states. Despite these issues, there is progress in this direction. Recent experiments reported the appearance of the spectral signatures, suggesting how the dark-type state participates and dictates the efficiency of energy transfer in the light-harvesting complex (LH2) from purple bacteria^6^ and in artificial cyanine nanotubes.^7^ Here, we employ theory to determine the role and suggest the implicit signatures of low-lying dark-type states in chlorosomes.

**Figure 1:**
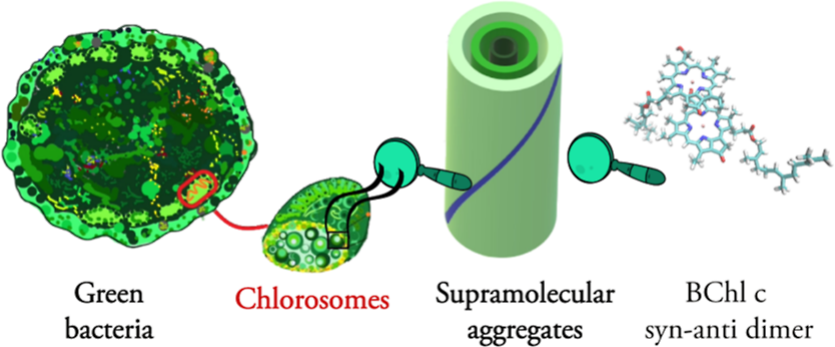
Chlorosomes are organelles responsible for light capture and initial energy transfer in green sulfur bacteria. Every chlorosome consists of secondary structures such as cylinders and lamellae. Our chlorosome model system consists of three concentric cylinders built from 27,829 BChl *c* molecules that form syn-anti parallel stacks.

The green sulfur bacteria *Chlorobaculum tepidum* are organisms that can perform photosynthesis in very low-light conditions, such as deep down in hot springs, at the bottom of the ocean,^8^ or using only geothermal energy.^9^ Its photosynthetic apparatus comprises chlorosomes, baseplate, Fenna–Matthews–Olson complex, and the reaction center.^2^ The energy flow through its photosynthetic apparatus has been studied in the 2D electronic spectroscopy (2DES) experiment^10^ and theoretically.^11,12^ This study will focus on chlorosomes, the organelles that perform the initial light capture and energy transfer in green bacteria. Their unique structure is an ensemble of supramolecular aggregates in cylindrical and lamellar shapes consisting predominantly of bacteriochlorophyll (BChl) *c* molecules (see Figure 1). Due to the heterogeneity of chlorosomes,^13^ various experimental techniques, combined with theoretical methods, provide information on their structure.^14−17^ In particular, nuclear magnetic resonance (NMR) spectroscopy,^15^ cryo-EM,^14,18^ soft-neutron scattering,^16^ microscopy,^17^ and optical spectroscopies^13,19,20^ have proven helpful.

There is a long history of optical spectroscopic studies that characterized the broad absorption band of chlorosomes. Absorption, linear and circular dichroism,^21^ and hole-burning^22,23^ experiments performed on the ensemble and several single molecule studies revealed the complexity of the optical band, which consists of distinct domains.^13,19,20^ Time-resolved spectroscopy studies offered insights into energy funneling through the photosynthetic apparatus,^10^ from chlorosomes to the baseplate,^24−26^ and within chlorosomes.^23,27,28^ These studies showed that energy transfer consists of different dynamical components with time scales spanning from hundreds of femtoseconds (10^–15^ s) to hundreds of picoseconds (10^–12^ s).^10,12^ A recent femtosecond transient absorption study connected different optical components in chlorosomes with distinct exciton transfer pathways (time scales) to the baseplate.^24^ 2DES experiments on isolated chlorosomes observed ultrafast transfer occurring within the initial sub-100 fs lifetime and the presence of quantum beatings with the ps lifetime.^29−33^ Due to the femtosecond time resolution, 2DES experiments are well suited to characterize exciton and charge dynamics in different materials, especially light-harvesting antennae.^34−36^ Such pump–probe, transient absorption, and 2DES experiments have previously successfully been applied to study ultrafast energy transfer in artificial J-aggregates.^37−39^ With pulse shaping and polarization sequence control, it is possible to probe distinct degrees of freedom by suppressing specific signals.^40^

Here, we will use a quantum-classical approach^41,42^ to simulate the 2DES spectra of a single chlorosome cylinder up to 500 fs waiting time and compare the results to experimental observations.^29^ Since we use an atomistic model^43^ of chlorosomal supramolecular aggregates^18,44,45^ to simulate spectroscopic observables,^46,47^ we can provide information on the molecular mechanisms behind ultrafast exciton transfer in chlorosomes without relying on fitting or on a prior assumption of a specific mechanism. We identify spectral signatures in the 2DES spectra of chlorosomes that arise due to the participation of low-lying dark states^48^ in the exciton dynamics. Additionally, we connect the presence of molecular disorder and fluctuations to the emergence of low-lying dark-type states in chlorosomes. Their presence agrees with the observation of the contribution of low-lying states with small transition dipole moments to optical spectra in a hole-burning study.^23^

It is important to note that due to the high computational cost of 2DES simulations, we calculated the spectral dynamics of a small system consisting of 2675 molecules embedded in the environment of a larger triple tube system. We evaluated the exciton parameters for a system of three concentric cylinders with 27,829 molecules. Such a large system size allows us to estimate statistical distributions of excitonic parameters that reflect the presence of molecular-scale disorder. The agreement between the absorption spectra of these two systems shows that the chosen small model is a good candidate for performing 2DES calculations, as shown previously (Figure S4 in ref (47)).

The rest of this article is organized as follows. First, we present our method of simulating the 2DES spectra of single chlorosome aggregate tubes based on the exciton model. Then we present the results of these simulations and their analysis, which uncover the presence of low-lying dark-type states in our model. We further characterize the nature of these states and show their contribution and importance for ultrafast exciton dynamics in chlorosomes. Finally, we present our conclusions and ideas for future research.

## Methods

The current work builds on a model developed and refined in multiple steps in refs (18,44,46,47,49). We will focus on the Q_*y*_ band^21^ arising from the strongly coupled Q_*y*_ transitions of BChl *c* in chlorosomes and represent the excited states using a Frenkel exciton Hamiltonian accounting for both structural and energetic disorder^50^
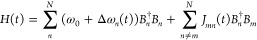
1

Here, *B*_*n*_^†^ and *B*_*n*_ are Paulionic creation and annihilation operators. This model depends on the sums over two key variables: transition energies ω_0_ + Δω_*n*_(*t*) and excitonic couplings *J*_*mn*_(*t*). ω_0_ of 15390 cm^–1^ is the energy gap for the Q_*y*_ transition of monomeric BChl *c* molecule in methanol,^21^ and Δω_*n*_(*t*) includes the effect of the electrostatic interactions with other molecules in the environment. The disorder in excitonic variables is based on the parameters from quantum chemistry studies on BChl *c* molecules in vacuum^46^ and molecular dynamics simulations of three concentric cylinders (Figure 1) representing chlorosomes.^44,46,46^ The details of the molecular dynamics simulation and quantum chemistry calculations were reported elsewhere.^46,47^ The main building blocks of these cylinders are syn-anti parallel stacks of BChl *c* molecules.^15^ Dispersion in the transition energies comes from differences in the electrostatic potential generated by partial charges of atoms in the environment that alter the transition energy gaps.^51^ We determined these dynamic energy gap fluctuations, Δω_*n*_(*t*), using the charge density approach.^51,52^ The excitonic coupling *J*_*mn*_ was determined using the point dipole approximation^53^ with a 5.48 Debye^21^ transition dipole moment, centered at magnesium and oriented between the nitrogens defining the *y*-axis of the BChl *c* molecules. Close packing of chromophores within chlorosomes challenges this approximation, but it was found to give good results in previous studies on helical cylindrical aggregates and chlorosome models.^18,20,54,55^ Since the Hamiltonian parametrically depends on the molecular dynamics simulations, we include the effects of static and dynamic disorder from classical intermolecular vibrations on the exciton dynamics. Due to the high computational costs coming from the large size of our system, we neglect the effects of intramolecular vibrations that are high-energy and localized. Additional justification is that very delocalized excitons in cylindrical systems are robust to local perturbations;^56^ however, such vibrations may lead to extra broadening and an increase in absorption in the high-energy tail of the spectra.

Previously, we showed a strong influence of the helicity of supramolecular aggregates on the excitonic structure, optical response, and ultrafast dynamics.^47^ Here, we will use a model of a chlorosomal cylinder with the helicity of wild-type chlorosomes, with a chiral angle of δ = 112.3°.^20,47^ This model structure’s optical properties align with the observations for chlorosomes from *C. tepidum* grown in low-light conditions. In this system, the mean of the distribution of angles between the transition dipole moment vectors of individual molecules and the long axis of the cylinder is β = 54°. Here, the exciton states have an overall approximately isotropic distribution of the direction of transition dipole moments, i.e., the excitonic transition dipole moments span all possible angles with the long axis of the cylinder.^47^

The time evolution of the exciton states is calculated using the NISE code^41,42,57^ that solves the time-dependent Schrödinger equation numerically
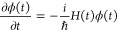
2

The main requirement of this method is for the Hamiltonian to be time-independent during a single time step Δ*t*. Hence, partitioning of Hamiltonian trajectory in short consecutive time steps allows propagating the wave function for longer times. In the case of chlorosomes, the time step was set to Δ*t* = 4 fs. During such a short time interval, nuclear dynamics can be neglected.^42^ Our quantum-classical approach represents a nonadiabatic simulation, where coupling between exciton states is driven by the bath fluctuations, as described by molecular dynamics simulation.^41,58^ This approach is a high-temperature approximation, which can influence the observed dynamics since it results in equal probabilities for populating all states in the eventual equilibrium state. Still, for the ultrafast events happening during hundreds of fs, NISE agrees with more exact approaches.^58^ We calculated the 2DES spectra using the perturbative response function formalism.^59^ Here, we focus on the all-parallel polarization pulse sequence.^29,60^ Our calculations are in the impulsive limit, hence, neglecting pulse shape effects. The coherence times (*t*_1_ and *t*_3_) are varied in the time interval [0, 196] fs with a time step δ*t* = 4 fs, while the population *t*_2_ time is calculated in the interval [0, 500] fs with time intervals around 24 fs. Due to the computationally expensive cubic scaling (*N*^3^) of the simulation with the number of molecules (*N*),^57,61^ we calculated the 2DES spectra of a single cylinder with 2675 BChl *c* molecules. More detailed information on the construction of this system was already provided in ref (47). Here, we averaged the 2DES spectral response over ten different realizations along the 10 ps long molecular dynamics trajectory, which causes the presence of some remnant structure in the simulated spectra, which would disappear with more extensive averaging. However, each realization of a 2D correlation map (ω_1_, ω_3_) per *t*_2_ time costs around 23,000 CPU hours on a supercomputer, deeming more averaging prohibitively expensive. To smoothen the calculated spectra, we convoluted the spectrum with the Lorentzian apodization function with a τ = 300 fs lifetime,^46^ which is equivalent to adding a small amount of homogeneous broadening.

## Results

**Figure 2:**
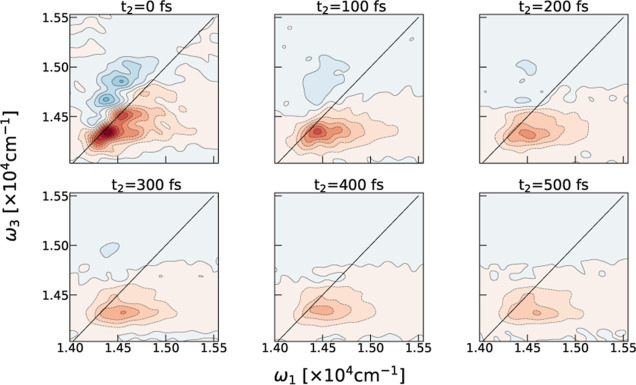
Time evolution of the calculated parallel polarization 2DES of a single chlorosome cylinder during the initial 500 fs.

2DES spectroscopy yields a complex signal of a third-order nonlinear response of the system.^62^ The 3D data set *I*_2DES_ (ω_1_, ω_3_, and *t*_2_) can be represented as an evolution of 2D correlation maps between pump (ω_1_) and probe (ω_3_) frequencies during a waiting time (*t*_2_). Figure 2 shows the time evolution of the calculated absorptive 2DES signal of the chlorosome model during the initial 500 fs.

The line shape changes in the signal reflect the dynamics in the system following the interaction with the initial laser pulses. The spectral signal consists of contributions from several processes such as depletion of the population in the ground state, i.e., ground-state bleach (GSB), stimulated emission (SE) from the single excited state, and excited state absorption (ESA), which leads to the simultaneous presence of two excitons in the system, i.e., doubly excited states. 2D correlation maps (ω_1_, ω_3_) for a specific *t*_2_ time show the contribution of the described dynamical processes. The bleach (red) features predominantly arise from the processes involving single excitations (GSB and SE), and absorption (blue) peaks quantify the double excitation dynamics (ESA). Thus, these maps capture the contribution of the ground state, single, and double-excitation manifolds to the observed dynamics. The spectral overlap and interference complicate the interpretation of the experimental results. Still, changes in the line shapes and peak-tilting serve as spectral signatures of energy transfer occurring in different light-harvesting systems,^3^ including chlorosomes.^29,32^ Our modeling confirms that the large width of the GSB and SE peak at *t*_2_ time arises from structural inhomogeneities on the molecular scale,^46^ as suggested previously.^22,23,29^ In line with the experiments,^29,30^ ultrafast exciton transfer will already happen during the initial 100 fs. Our model confirms that the observed ultrafast dynamics can occur within a single chlorosome cylinder. This process modifies spectral signals by rounding the line shapes due to memory loss^40^ and peak tilting toward the lower probe frequencies following the downhill energy transfer.^29^ Large exciton delocalization^46^ ensures this process’ fast rate and robustness. Despite the disorder-induced localization of excitons^63,64^ on specific segments within a cylindrical aggregate,^29^ the close packing of chromophores and the extended nature of exciton coupling due to the cylindrical structure ensure the delocalization of bright excitons over hundreds of molecules.^46,65^ Bath fluctuations lead to nonadiabatic coupling between exciton states, which drives ultrafast relaxation within the exciton band. The robustness of this energy transfer comes from many delocalized exciton states within a single cylinder, which are close in energy. Therefore, classical low-energy fluctuations can induce exciton state mixing on the ultrafast time scale. Our exciton simulations are performed in the infinite temperature limit, which affects the simulated dynamics through the increased probability of populating higher energy states. Still, we recovered all the key features of the experiment.^29^ Large system size, good behavior of the NISE method on the ultrafast time scale, compared to a numerically exact method,^58^ and a good agreement with the experiment^29^ support our choice of the method.

**Figure 3:**
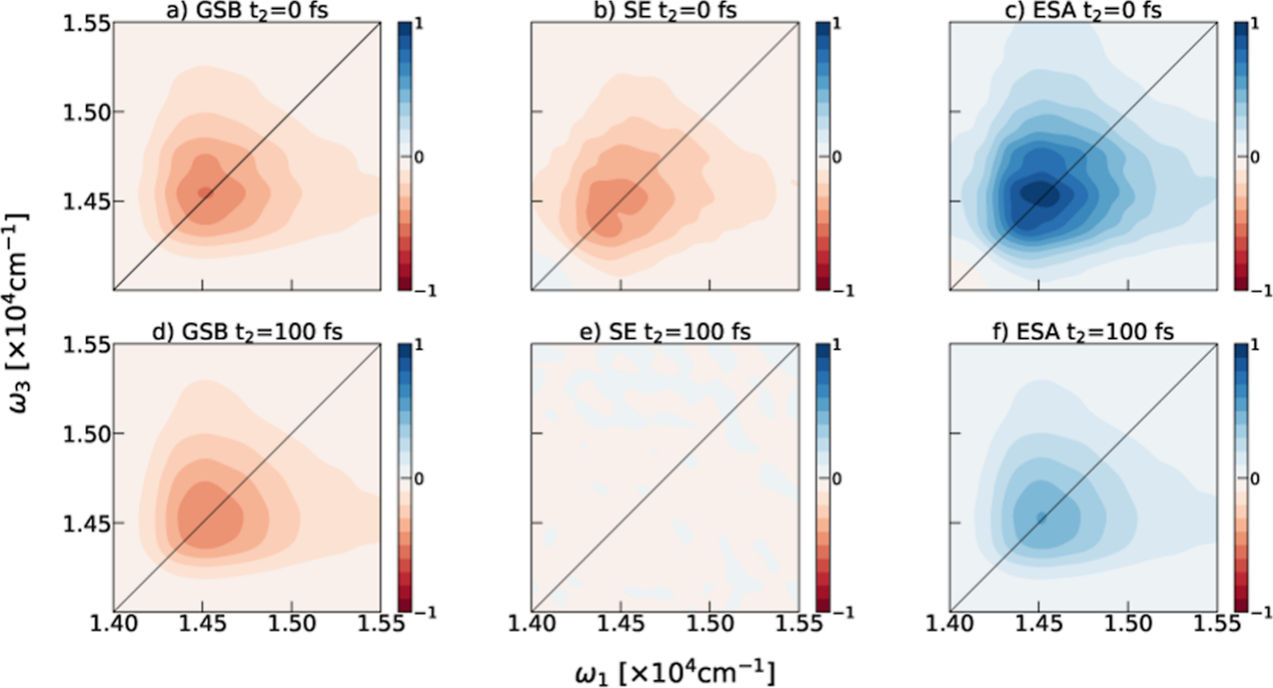
2D correlation maps show signals of the GSB, SE, and ESA. The spectra on the top panel (a–c) show the contribution of these processes to the 2DES spectra of chlorosomes at *t*_2_ = 0 fs waiting time. The spectra on the bottom (d,f,g) are for *t*_2_ = 100 fs waiting time. We normalized all signals to the point of maximum intensity of the total 2DES spectra at *t*_2_ = 0 waiting time.

To characterize how the different dynamical processes affect observed line shape changes and to separate ground from excited state dynamics, we separated the contributions from GSB, SE, and ESA to the overall signal. Here, we focus the discussion on results at *t*_2_ = 0 fs and *t*_2_ = 100 fs, as shown in Figure 3.

The GSB signal exclusively reports on the dynamics of the ground-state vibrations included in our model. It does not depend on population transfer. The ESA signal depends on the dynamics in the exciton landscape and population transfer. Therefore, line shape differences observed between these signals arise from different dynamic processes. Still, we see that GSB and ESA contributions mostly overlap both at *t*_2_ = 0 fs and later at *t*_2_ = 100 fs. This overlap results in interference between the signals, leading the diagonal peak to be pushed below the diagonal, as shown in Figure 2. Both signals persist during the first 100 fs and exhibit significant rounding of the peaks, reflecting the loss of correlation. As discussed previously, the GSB signal reveals memory loss in the bath, while the ESA signal also captures the loss of correlation due to energy transfer during the population time. The SE signal contribution for *t*_2_ = 0 time contains two subpeaks, which lie lower and higher from the maximum point of the GSB and ESA signal (see Section 3). Due to the arrangement of BChl *c* molecules within the chlorosome model, we observe a very dispersed distribution of transition dipole moment orientations of exciton states emerging in this system.^47^ We connect the two different peaks in the SE signal with peak splitting of the absorption components, showing exciton states with transition dipole moments perpendicular to the long axis of the cylinder. We note that the system size determines the presence and position of these states, and their resolution vanishes for large aggregates, in line with previous findings for cylindrical aggregates, as shown in Figure S5 in ref (47). We note that such a peak splitting arises from the strong dependence of the perpendicular exciton states on the radius of the cylinders.^66^ Since these signals do not appear in the experimental spectra,^29^ they could be signatures of finite-size effects^67^ or can be unresolved in the presence of a large number of cylinders, and even lamellae, in the chlorosomal organelle, i.e., due to significant mesoscale disorder.^13,22^ With this, we explain the peak splitting in the overall 2DES spectra at *t*_2_ = 0 fs time, as observed for both the blue and red signals shown in Figure 2. With this, we confirm that higher-lying points on the diagonal, corresponding to large (ω_1_, ω_3_), are free from GSB dynamics, so the ESA and SE processes predominantly contribute to the signal. Notably, the SE contribution completely decays on a sub-100 fs time scale, reflecting the lifetime of the bright single excited states. As the energy dissipation from the single exciton manifold is not part of our dynamics simulations, this must be because the system reaches excited states which do not contribute to the SE process. The longer lifetime of the ESA component also suggests the presence of electric-dipole-forbidden single exciton states that still can get promoted to the double-excited state manifold by the probe pulse. Hence, we identify this as a spectral signature of low-lying dark-type exciton states in chlorosomes, which do not contribute to coherent emission. This conclusion agrees with findings from the hole-burning experiments, which indicated the existence of a low-energy spectral band with a small transition dipole moments^23^ and significant fluorescence quenching.^68^ The decomposition of the total 2DES spectra in separate contributions is not possible solely from the experimental data, which emphasizes the importance of computational modeling for deciphering the dynamics behind these complex signals. Additionally, 2D fluorescence-excitation (2D-FLEX) spectroscopy, the recently proposed technique which probes only excited state dynamics,^69^ could directly detect ultrafast decay of the SE signal in chlorosomes.

**Figure 4:**
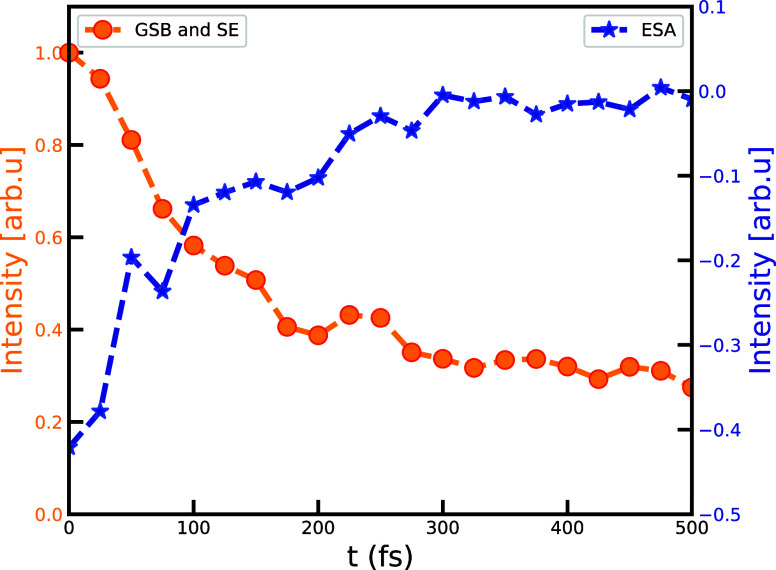
Time evolution of the point with minimal (negative) signal *I*_min_ (ω_1_ = 14,397 cm^–1^, ω_3_ = 14,344 cm^–1^) (orange) and maximal positive signal *I*_max_ (ω_1_ = 14,377 cm^–1^, ω_3_ = 14,670 cm^–1^) (blue). Each trace has its own *y*-axis for the ease of comparison.

The experimental observation of a long-lived ESA peak, which behaves largely as a mirror image of the evolution of the GSB/SE peak,^30^ supports our findings. Hence, we report the simulated time evolution of the two points *I*_min_ (ω_1_ = 14397 cm^–1^, ω_3_ = 14344 cm^–1^) and *I*_max_ (ω_1_ = 14377 cm^–1^, ω_3_ = 14670 cm^–1^) in Figure 4.

We observe oscillations in these traces that persist until 500 fs. The decay of the SE signal within the initial 100 fs rules out the possibility that these beatings come from electronic coherence. Hence, these come from low-frequency classical vibrations. We will not further analyze the nature of these vibrations. Instead, we will discuss the conditions that lead to the emergence of low-lying dark-type exciton states in chlorosomes and characterize their nature.

**Figure 5:**
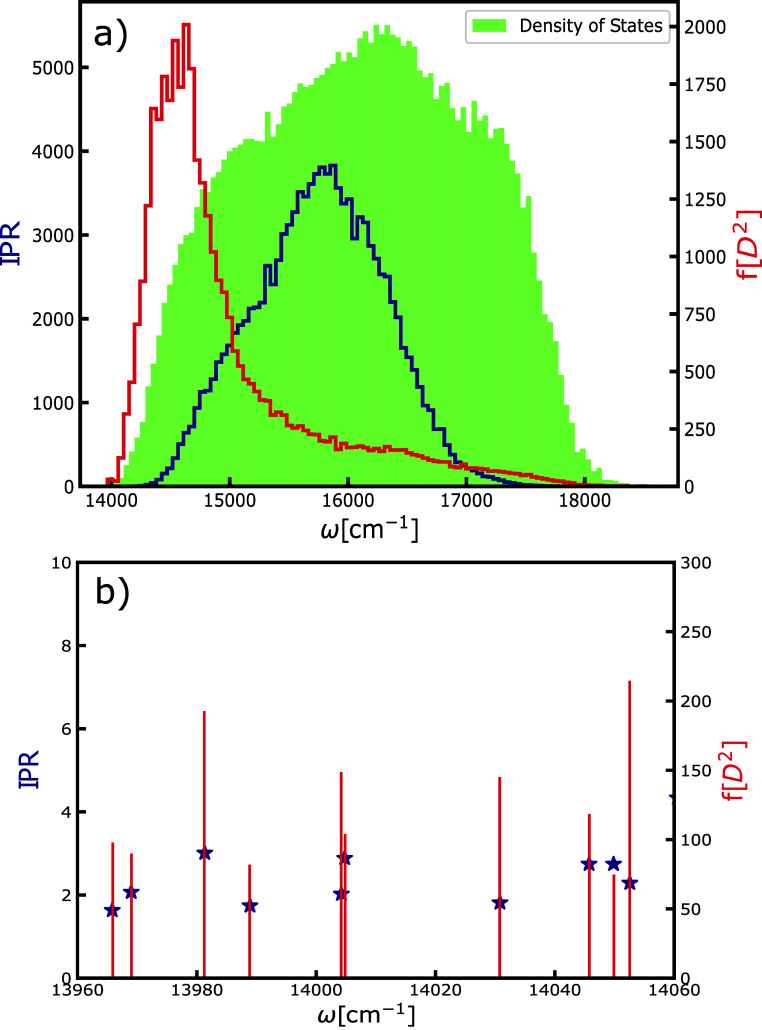
(a) Histogram of the DOS (in green), with the inverse participation ratio (dark blue) and oscillator strength (red) as the functions of exciton energy. From here, we can observe the presence of low-lying localized (dark-type) excitons. These states are found in the lower edge of the exciton band. (b) Representation of the ten lowest-lying exciton states in one simulation frame of the chlorosome system. Red sticks represent the oscillator strength (*f* = μ^2^), and blue stars represent the values of the inverse participation ratio. The states with the weakest signals have oscillator strength ∼40× weaker than the strongest-superradiant states. These states delocalize over at least two molecules, confirming their multichromophoric character.

Figure 5 shows the histogram of the density of states (DOS), plots of the inverse participation ratio, and oscillator strength within the single-exciton band.

Optically active excitons are near the lower edge of the band, as generally observed for J-aggregates.^70^ The figure also shows how the delocalization of exciton states grows until the middle of the band, after which it decreases, exhibiting localization-delocalization crossover,^46,71^ as typically occurs in cylindrical structures. States on the band edge are the most sensitive to the presence of disorder.^70^ Most importantly, we observe the low-lying, localized states with small transition moments and thus low oscillator strength. The intensity of their signals is ∼40 to 50 times weaker compared with the brightest superradiant states in chlorosomes. Importantly these dark-type states are thus not absolutely dark but orders of magnitude weaker than the superradiant states responsible for the absorption. Here, we observe a distribution and not a single dark-type exciton state, in line with observations from hole-burning experiments.^23^ In summary, compared to the superradiant exciton states in chlorosomes, the identified low-lying dark-type states do not contribute to the stimulated (coherent emission) response since they have significantly weaker signals. Also, these low-lying states are multichromophoric, as observed for peridinin–chlorophyll–protein.^72^ We see that these states delocalize over at least two molecules due to the close packing of BChl molecules into syn-anti parallel stacks.^15^ This delocalization could also explain the observation of collective spontaneous emission in chlorosomes from states delocalized over two molecules.^68^ Still, the radiative lifetime of these states is estimated to be around 18.4 ps,^68^ which is significantly longer than our simulation time, so it does not contribute to the sub-500 fs dynamics. Low-lying dark-type states were also observed for several linear^48^ and cylindrical^65^ molecular aggregates.

**Figure 6:**
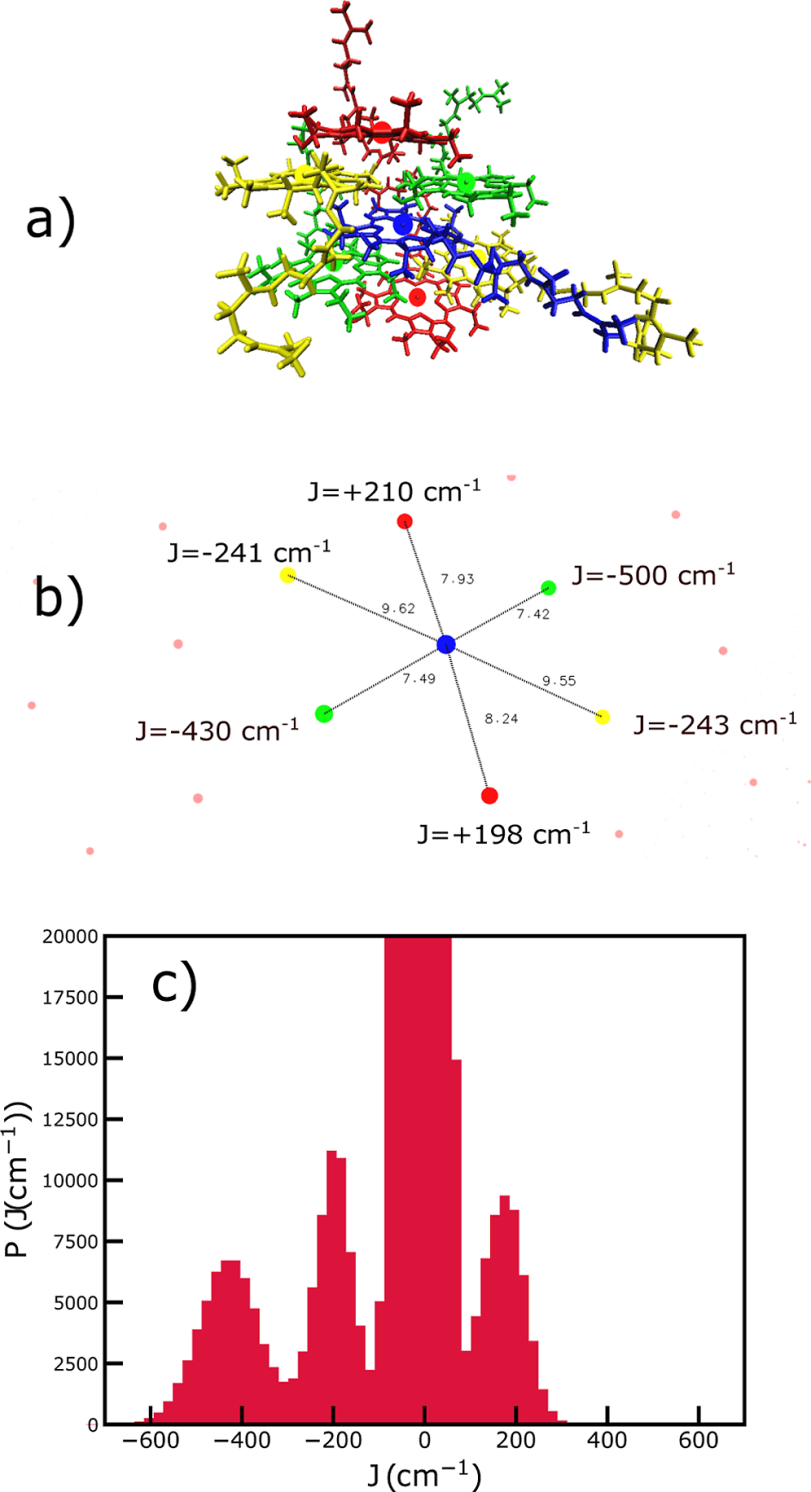
(a) Illustration of the local structural environment around a representative central BChl *c* molecule (blue) surrounded by neighbors in green, yellow, and red. The nearest neighbors, with the strongest couplings, are in the same syn-anti stack (green molecules), while the red and yellow molecules are in the neighboring stacks. (b) Example of couplings between the central blue molecule and its neighbors shown by highlighting the magnesium positions. The Mg–Mg distances are given in Ångstrøm. More distant magnesium atoms are shown as pink dots. (c) Distribution of the exciton couplings (red) calculated for the initial structure of the chlorosome tube. The large peak around zero, which goes far beyond the scale of the *y*-axis, shows the presence of a large number of distant pairs of weakly coupled molecules. Other peaks correspond to the nearest-neighbor and next-nearest-neighbor couplings.

Recent studies on 2D systems showed that different signs of exciton couplings in distinct spatial directions lead to changes in the DOS and optical activity.^73,74^ To further understand the coupling behavior in chlorosomes, we show the distribution of exciton couplings in chlorosomes in the lower panel of Figure 6.

Multiple peaks in this distribution reveal the structural packing on the local scale and show three spatial directions in which the molecules have strong exciton coupling. The molecules have some positive but predominantly negative couplings, leading to the experimentally observed overall red shift of the chlorosome (Q_*y*_ band) compared to the monomeric BChl *c* absorption spectra.^26^ However, due to the positive exciton couplings, the brightest absorption lines are not at the total bottom of the band, distinguishing chlorosomes from a typical picture of J-aggregates. The structural arrangement of the molecules, illustrating the origin of different couplings, is shown in Figure 6. The negative coupling peak centered around 450 cm^–1^ represents the nearest-neighbor interaction within syn-anti parallel stacks. The propagation of these stacks creates helical arrangements within chlorosomes.^15,49^ The two peaks centered around +200 and −200 cm^–1^ give two different kinds of next-nearest-neighbor interactions. These come from the interactions with the BChl *c* molecules from the syn-anti dimers in the neighboring helices. A broad distribution of excitonic couplings that includes positive and negative values, combined with the dispersion in the transition energies due to differences in the electrostatic environments around each BChl *c* molecule within the aggregates,^46^ leads to the dispersion of the oscillator strength within the exciton band^54^ and the emergence of the low-lying dark-type exciton states. These states can act as collector states^75^ in light-harvesting antennae.

**Figure 7:**
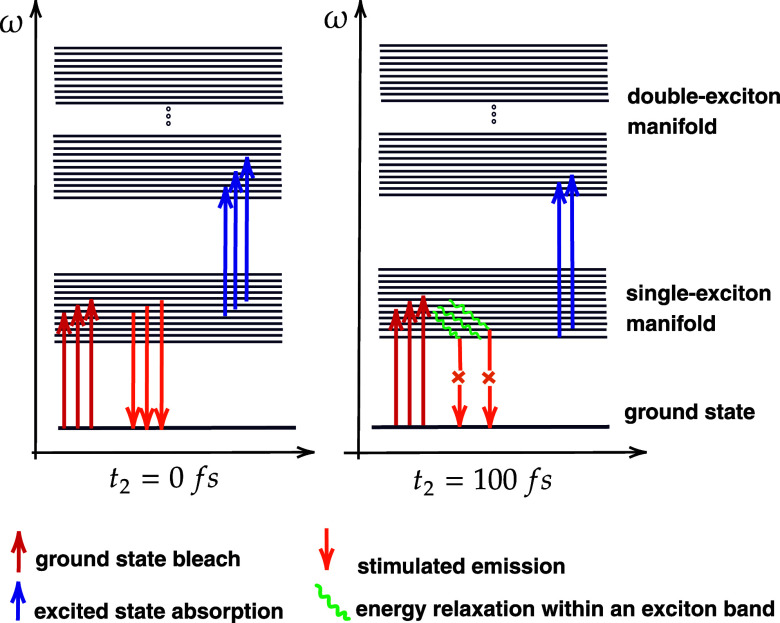
Jablonski diagrams compare dynamical processes captured in the 2DES spectra of chlorosomes at *t*_2_ = 0 fs and *t*_2_ = 100 fs waiting time.

Now, we can present the connection between the low-lying dark-type exciton states and the spectral changes in the 2DES spectra, as illustrated in Figure 7.

In the initial *t*_2_ of 0 fs, pump pulses excite bright excitons. A probe pulse interacts with these states, which gives the SE contribution to the total 2D correlation map at *t*_2_ = 0 fs. For longer waiting times, such as *t*_2_ = 100 fs, the bright exciton states will relax within the band toward the lower-lying states. The sub-100 fs relaxation relies on bath fluctuations that mix exciton states through nonadiabatic couplings. As we showed, these states are dark-type, so their connection to the ground state is magnitudes lower than that of the superradiant states, and so they do not contribute to the SE signal at *t*_2_ = 0 fs. The estimated lifetime of bright excitons of around 100 fs agrees well with the homogeneous line width of 360 cm^–1^ in 2DES spectra reported in the experiments.^29^ Additionally, the simulated dynamics of 2D spectra for the maximum and minimum intensity points, as shown in Figure 4, is compatible with the experimental observation.^30^ These low-lying excitons contribute to the ESA signals. The large number of states in the double-excited state manifold allows for *N* – 1 possible connections for the single exciton to the double-exciton manifold. Hence, the probe pulse can promote dark-type excitons to the double-exciton manifold. The ESA signal is almost as strong as the GSB signal in the delocalized systems. We show that the experimentally observed long-living ESA signal^30^ presents a spectral signature of low-lying dark-type multichromophoric states in chlorosomes.

## Conclusions

This study deals with the nature and role of dark-type states in chlorosomes, providing a novel insight into one of the highlighted open questions on the mechanisms of photosynthesis.^5^ Starting from the atomistic structure of chlorosomes and molecular dynamics simulations,^44^ we constructed a disordered Frenkel exciton model and computed the dynamics of the 2D electronic spectra up to a delay time of 500 fs. Our simulations capture the ultrafast dynamics in a single chlorosome aggregate. We observe significant changes in the line shapes and rounding of the peaks within the first 100 fs, in line with the experiments.^29,30^

2DES experiments measure a total signal from which it is not directly possible to separate the effects of different dynamic processes, signifying the importance of explicit simulation of the contributions of the GSB, SE, and ESA processes and separating the excited state from the ground-state dynamics. The decay of the SE signal on a sub-100 fs time scale shows complete relaxation from the initially excited bright states within the exciton band to dark-type states. The probe pulse can promote these dark states to the double-excited state manifold and contribute to the ESA signal. While experiments reported this dynamic component,^23,29^ they omitted its assignment as the energy transfer to the low-lying dark exciton states. Our results show that the brightest states, responsible for light absorption, are not significant in the energy transfer processes to the baseplate, which are slower than the observed sub-100 fs time scale. This provides new insights into the mechanisms responsible for the efficiency of the function of chlorosomes.

Dark states participate in and dominate the ultrafast exciton dynamics occurring in a single chlorosome aggregate after 100 fs. Through the population of dark-type states, energy loss due to radiative processes, such as re-emission, is effectively suppressed. Their longer lifetime ensures that energy can be efficiently transferred to the next complex,^75^ here the baseplate.^24,26^ Similar dark-type exciton states are also present on the band edge of the single-walled carbon nanotubes,^76^ with comparable time scales of hundreds of femtoseconds of relaxation of high-lying bright to low-lying dark-type excitons, quantum dots,^77^ and other molecular aggregates^7,65^ and light-harvesting antennae.^5,6^

We show how molecular disorder alters the exciton landscape and redistributes the oscillator strength over many exciton states, leading to the emergence of low-lying dark-type exciton states in chlorosomes. These states are multichromophoric, and they delocalize over at least two molecules. Thus, we connect the multichromophoric low-lying exciton states with the spectral signature that suggests their presence. Our study focuses on isolated chlorosomes, raising the question of how probable funneling through this energy pathway is in the whole photosynthetic apparatus of green sulfur bacteria.^10,78^ The longer lifetime of the dark-type states can support efficient energy transfer to the baseplate, the next light-harvesting complex in the photosynthetic apparatus of green sulfur bacteria, occurring on a picosecond time scale,^24^ without the risk of radiative energy losses to which bright states are susceptible. On the other hand, relaxation to the dark-type states may compete with other pathways toward the baseplate. Further investigations of the chlorosome-baseplate system would provide valuable insights into the role of dark-type states in energy transfer in green sulfur bacteria.

## References

[ref1] Blankenship R. E. Molecular Mechanisms of Photosynthesis, 3rd ed.; Wiley: Oxford, U.K., 2021.

[ref2] Mirkovic T.; Ostroumov E. E.; Anna J. M.; Van Grondelle R.; Scholes G. D.; Scholes G. D. Light absorption and energy transfer in the antenna complexes of photosynthetic organisms. Chem. Rev. 2017, 117, 249–293. 10.1021/acs.chemrev.6b00002. 27428615

[ref3] Fiebig O. C.; Harris D.; Wang D.; Hoffmann M. P.; Schlau-Cohen G. S. Ultrafast Dynamics of Photosynthetic Light Harvesting: Strategies for Acclimation Across Organisms. Annu. Rev. Phys. Chem. 2023, 74, 493–520. 10.1146/annurev-physchem-083122-111318. 36791782

[ref4] Saga Y.; Shibata Y.; Tamiaki H. Spectral properties of single light-harvesting complexes in bacterial photosynthesis. J. Photochem. Photobiol. C. Photochem. Rev. 2010, 11, 15–24. 10.1016/j.jphotochemrev.2010.02.002.

[ref5] Reimers J. R.; Biczysko M.; Bruce D.; Coker D. F.; Frankcombe T. J.; Hashimoto H.; Hauer J.; Jankowiak R.; Kramer T.; Linnanto J.; et al. Challenges facing an understanding of the nature of low-energy excited states in photosynthesis. Biochim. Biophys. Acta Bioenerg. 2016, 1857, 1627–1640. 10.1016/j.bbabio.2016.06.010. 27372198

[ref6] Ferretti M.; Hendrikx R.; Romero E.; Southall J.; Cogdell R. J.; Novoderezhkin V. I.; Scholes G. D.; Van Grondelle R. Dark states in the light-harvesting complex 2 revealed by two-dimensional electronic spectroscopy. Sci. Rep. 2016, 6, 20834–20839. 10.1038/srep20834. 26857477 PMC4746630

[ref7] Doria S.; Di Donato M.; Borrelli R.; Gelin M. F.; Caram J.; Pagliai M.; Foggi P.; Lapini A. Vibronic coherences in light harvesting nanotubes: unravelling the role of dark states. J. Mater. Chem. C 2022, 10, 7216–7226. 10.1039/D2TC00203E.

[ref8] Marschall E.; Jogler M.; Henßge U.; Overmann J. Large-scale distribution and activity patterns of an extremely low-light-adapted population of green sulfur bacteria in the Black Sea. Environ. Microbiol. 2010, 12, 1348–1362. 10.1111/j.1462-2920.2010.02178.x. 20236170

[ref9] Beatty J. T.; Overmann J.; Lince M. T.; Manske A. K.; Lang A. S.; Blankenship R. E.; Van Dover C. L.; Martinson T. A.; Plumley F. G. An obligately photosynthetic bacterial anaerobe from a deep-sea hydrothermal vent. Proc. Natl. Acad. Sci. U. S. A. 2005, 102, 9306–9310. 10.1073/pnas.0503674102. 15967984 PMC1166624

[ref10] Dostál J.; Pšenčík J.; Zigmantas D. In situ mapping of the energy flow through the entire photosynthetic apparatus. Nat. Chem. 2016, 8, 705–710. 10.1038/nchem.2525. 27325098

[ref11] Kramer T.; Rodriguez M. Two-dimensional electronic spectra of the photosynthetic apparatus of green sulfur bacteria. Sci. Rep. 2017, 7, 45245 10.1038/srep45245. 28345621 PMC5366913

[ref12] Huh J.; Saikin S. K.; Brookes J. C.; Valleau S.; Fujita T.; Aspuru-Guzik A. Atomistic study of energy funneling in the light-harvesting complex of green sulfur bacteria. J. Am. Chem. Soc. 2014, 136, 2048–2057. 10.1021/ja412035q. 24405318

[ref13] Furumaki S.; Vacha F.; Habuchi S.; Tsukatani Y.; Bryant D. A.; Vacha M. Absorption linear dichroism measured directly on a single light-harvesting system: the role of disorder in chlorosomes of green photosynthetic bacteria. J. Am. Chem. Soc. 2011, 133, 6703–6710. 10.1021/ja111475z. 21476570

[ref14] Oostergetel G. T.; Reus M.; Gomez Maqueo Chew A.; Bryant D. A.; Boekema E. J.; Holzwarth A. R. Long-range organization of bacteriochlorophyll in chlorosomes of Chlorobium tepidum investigated by cryo-electron microscopy. FEBS Lett. 2007, 581, 5435–5439. 10.1016/j.febslet.2007.10.045. 17981156

[ref15] Ganapathy S.; Oostergetel G. T.; Wawrzyniak P. K.; Reus M.; Gomez Maqueo Chew A.; Buda F.; Boekema E. J.; Bryant D. A.; Holzwarth A. R.; De Groot H. J. Alternating syn-anti bacteriochlorophylls form concentric helical nanotubes in chlorosomes. Proc. Natl. Acad. Sci. U. S. A. 2009, 106, 8525–8530. 10.1073/pnas.0903534106. 19435848 PMC2680731

[ref16] Tang K.-H.; Urban V. S.; Wen J.; Xin Y.; Blankenship R. E. SANS investigation of the photosynthetic machinery of Chloroflexus aurantiacus. Biophys. J. 2010, 99, 2398–2407. 10.1016/j.bpj.2010.07.068. 20959079 PMC2956220

[ref17] Tian Y.; Camacho R.; Thomsson D.; Reus M.; Holzwarth A. R.; Scheblykin I. G. Organization of bacteriochlorophylls in individual chlorosomes from Chlorobaculum tepidum studied by 2-dimensional polarization fluorescence microscopy. J. Am. Chem. Soc. 2011, 133, 17192–17199. 10.1021/ja2019959. 21923120

[ref18] Li X.; Buda F.; de Groot H. J.; Sevink G. J. A. Molecular insight in the optical response of tubular chlorosomal assemblies. J. Phys. Chem. C 2019, 123, 16462–16478. 10.1021/acs.jpcc.9b03913.

[ref19] Furumaki S.; Yabiku Y.; Habuchi S.; Tsukatani Y.; Bryant D. A.; Vacha M. Circular dichroism measured on single chlorosomal light-harvesting complexes of green photosynthetic bacteria. J. Phys. Chem. Lett. 2012, 3, 3545–3549. 10.1021/jz301671p. 26290985

[ref20] Günther L. M.; Löhner A.; Reiher C.; Kunsel T.; Jansen T. L. C.; Tank M.; Bryant D. A.; Knoester J.; Köhler J. Structural variations in chlorosomes from wild-type and a bchQR mutant of Chlorobaculum tepidum revealed by single-molecule spectroscopy. J. Phys. Chem. B 2018, 122, 6712–6723. 10.1021/acs.jpcb.8b02875. 29863357

[ref21] Prokhorenko V.; Steensgaard D.; Holzwarth A. Exciton theory for supramolecular chlorosomal aggregates: 1. Aggregate size dependence of the linear spectra. Biophys. J. 2003, 85, 3173–3186. 10.1016/S0006-3495(03)74735-3. 14581217 PMC1303593

[ref22] Pšenčíik J.; Vácha M.; Adamec F.; Ambro\>z M.; Dian J.; Bo\>cek J.; Hála J. Hole burning study of excited state structure and energy transfer dynamics of bacteriochlorophyll c in chlorosomes of green sulphur photosynthetic bacteria. Photosyn. Res. 1994, 42, 1–8. 10.1007/bf00019052. 24307462

[ref23] Pšenčík J.; Polívka T.; Němec P.; Dian J.; Kudrna J.; Malý P.; Hála J. Fast energy transfer and exciton dynamics in chlorosomes of the green sulfur bacterium Chlorobium tepidum. J. Phys. Chem. A 1998, 102, 4392–4398. 10.1021/jp973227y.

[ref24] Frehan S. K.; Dsouza L.; Li X.; Eríc V.; Jansen T. L.; Mul G.; Holzwarth A. R.; Buda F.; Sevink G. J. A.; de Groot H. J.; et al. Photon Energy-Dependent Ultrafast Exciton Transfer in Chlorosomes of Chlorobium tepidum and the Role of Supramolecular Dynamics. J. Phys. Chem. B 2023, 127, 7581–7589. 10.1021/acs.jpcb.3c05282. 37611240 PMC10493955

[ref25] Martiskainen J.; Linnanto J.; Kananavičius R.; Lehtovuori V.; Korppi-Tommola J. Excitation energy transfer in isolated chlorosomes from Chloroflexus aurantiacus. Chem. Phys. Lett. 2009, 477, 216–220. 10.1016/j.cplett.2009.06.080.

[ref26] Martiskainen J.; Linnanto J.; Aumanen V.; Myllyperkiö P.; Korppi-Tommola J. Excitation energy transfer in isolated chlorosomes from Chlorobaculum tepidum and Prosthecochloris aestuarii. Photochem. Photobiol. 2012, 88, 675–683. 10.1111/j.1751-1097.2012.01098.x. 22272813

[ref27] Savikhin S.; Zhu Y.; Lin S.; Blankenship R. E.; Struve W. S. Femtosecond spectroscopy of chlorosome antennas from the green photosynthetic bacterium Chloroflexus aurantiacus. J. Phys. Chem. 1994, 98, 10322–10334. 10.1021/j100091a056. 11539413

[ref28] Savikhin S.; van Noort P. I.; Zhu Y.; Lin S.; Blankenship R. E.; Struve W. S. Ultrafast energy transfer in light-harvesting chlorosomes from the green sulfur bacterium Chlorobium tepidum. Chem. Phys. 1995, 194, 245–258. 10.1016/0301-0104(95)00019-K. 11540594

[ref29] Dostal J.; Mancal T.; Augulis R.-n.; Vacha F.; Psencik J.; Zigmantas D. Two-dimensional electronic spectroscopy reveals ultrafast energy diffusion in chlorosomes. J. Am. Chem. Soc. 2012, 134, 11611–11617. 10.1021/ja3025627. 22690836

[ref30] Dostál J.; Mančal T.; Vácha F.; Pšenčík J.; Zigmantas D. Unraveling the nature of coherent beatings in chlorosomes. J. Chem. Phys. 2014, 140, 03B616_1 10.1063/1.4868557. 24655205

[ref31] Jun S.; Yang C.; Isaji M.; Tamiaki H.; Kim J.; Ihee H. Coherent oscillations in chlorosome elucidated by two-dimensional electronic spectroscopy. J. Phys. Chem. Lett. 2014, 5, 1386–1392. 10.1021/jz500328w. 26269984

[ref32] Jun S.; Yang C.; Kim T. W.; Isaji M.; Tamiaki H.; Ihee H.; Kim J. Role of thermal excitation in ultrafast energy transfer in chlorosomes revealed by two-dimensional electronic spectroscopy. Phys. Chem. Chem. Phys. 2015, 17, 17872–17879. 10.1039/C5CP01355K. 26095203

[ref33] Jun S.; Yang C.; Choi S.; Isaji M.; Tamiaki H.; Ihee H.; Kim J. Exciton delocalization length in chlorosomes investigated by lineshape dynamics of two-dimensional electronic spectra. Phys. Chem. Chem. Phys. 2021, 23, 24111–24117. 10.1039/D1CP03413H. 34498018

[ref34] Jonas D. M. Two-dimensional femtosecond spectroscopy. Annu. Rev. Phys. Chem. 2003, 54, 425–463. 10.1146/annurev.physchem.54.011002.103907. 12626736

[ref35] Biswas S.; Kim J.; Zhang X.; Scholes G. D. Coherent two-dimensional and broadband electronic spectroscopies. Chem. Rev. 2022, 122, 4257–4321. 10.1021/acs.chemrev.1c00623. 35037757

[ref36] Collini E. 2D electronic spectroscopic techniques for quantum technology applications. J. Phys. Chem. C 2021, 125, 13096–13108. 10.1021/acs.jpcc.1c02693. PMC8282191 34276867

[ref37] Lee J.-H.; Min C.-K.; Joo T. Ultrafast optical dynamics of excitons in J-aggregates. J. Chem. Phys. 2001, 114, 377–381. 10.1063/1.1329133.

[ref38] Fang H.; Wilhelm M. J.; Kuhn D. L.; Zander Z.; Dai H.-L.; Petersson G. A. The low-lying electronic states and ultrafast relaxation dynamics of the monomers and J-aggregates of meso-tetrakis (4-sulfonatophenyl)-porphyrins. J. Chem. Phys. 2023, 159, 154302 10.1063/5.0174368. 37846956

[ref39] Bolzonello L.; Fassioli F.; Collini E. Correlated fluctuations and intraband dynamics of J-aggregates revealed by combination of 2DES schemes. J. Phys. Chem. Lett. 2016, 7, 4996–5001. 10.1021/acs.jpclett.6b02433. 27973862 PMC5165657

[ref40] Hamm P.; Zanni M. Concepts and Methods of 2D Infrared Spectroscopy; Cambridge University Press, 2011.

[ref41] Jansen T. L. C. Computational spectroscopy of complex systems. J. Chem. Phys. 2021, 155, 170901 10.1063/5.0064092. 34742221

[ref42] Jansen T. L. C.; Knoester J. Nonadiabatic effects in the two-dimensional infrared spectra of peptides: application to alanine dipeptide. J. Phys. Chem. B 2006, 110, 22910–22916. 10.1021/jp064795t. 17092043

[ref43] Cignoni E.; Slama V.; Cupellini L.; Mennucci B. The atomistic modeling of light-harvesting complexes from the physical models to the computational protocol. J. Chem. Phys. 2022, 156, 120901 10.1063/5.0086275. 35364859

[ref44] Li X.; Buda F.; de Groot H. J.; Sevink G. J. A. Contrasting modes of self-assembly and hydrogen-bonding heterogeneity in chlorosomes of Chlorobaculum tepidum. J. Phys. Chem. C 2018, 122, 14877–14888. 10.1021/acs.jpcc.8b01790. PMC6150686 30258522

[ref45] Li X.; Buda F.; de Groot H. J.; Sevink G. J. A. Dynamic disorder drives exciton transfer in tubular chlorosomal assemblies. J. Phys. Chem. B 2020, 124, 4026–4035. 10.1021/acs.jpcb.0c00441. 32343578 PMC7246976

[ref46] Erić V.; Li X.; Dsouza L.; Frehan S. K.; Huijser A.; Holzwarth A. R.; Buda F.; Sevink G. J. A.; de Groot H. J.; Jansen T. L. C. Manifestation of Hydrogen Bonding and Exciton Delocalization on the Absorption and Two-Dimensional Electronic Spectra of Chlorosomes. J. Phys. Chem. B 2023, 127, 1097–1109. 10.1021/acs.jpcb.2c07143. 36696537 PMC9923760

[ref47] Erić V.; Castro J. L.; Li X.; Dsouza L.; Frehan S. K.; Huijser A.; Holzwarth A. R.; Buda F.; Sevink G. J. A.; de Groot H. J.; Jansen T. Ultrafast Anisotropy Decay Reveals Structure and Energy Transfer in Supramolecular Aggregates. J. Phys. Chem. B 2023, 127, 7487–7496. 10.1021/acs.jpcb.3c04719. 37594912 PMC10476209

[ref48] Pugžlys A.; Augulis R.; Van Loosdrecht P.; Didraga C.; Malyshev V.; Knoester J. Temperature-dependent relaxation of excitons in tubular molecular aggregates: fluorescence decay and Stokes shift. J. Phys. Chem. B 2006, 110, 20268–20276. 10.1021/jp062983d. 17034206

[ref49] Li X.; Buda F.; de Groot H. J.; Sevink G. J. A. The role of chirality and plastic crystallinity in the optical and mechanical properties of chlorosomes. iScience 2022, 25, 103618 10.1016/j.isci.2021.103618. 35005556 PMC8719020

[ref50] Davydov A. S. The Theory of Molecular Excitons. Sov. Phys. Usp. 1964, 7, 145–178. 10.1070/PU1964v007n02ABEH003659.

[ref51] Madjet M.; Abdurahman A.; Renger T. Intermolecular Coulomb couplings from ab initio electrostatic potentials: application to optical transitions of strongly coupled pigments in photosynthetic antennae and reaction centers. J. Phys. Chem. B 2006, 110, 17268–17281. 10.1021/jp0615398. 16928026

[ref52] Renger T.; Madjet M. E.-A.; Schmidt am Busch M.; Adolphs J.; Müh F. Structure-based modeling of energy transfer in photosynthesis. Photosyn. Res. 2013, 116, 367–388. 10.1007/s11120-013-9893-3. 23921525

[ref53] Didraga C.; Klugkist J. A.; Knoester J. Optical properties of helical cylindrical molecular aggregates: the homogeneous limit. J. Phys. Chem. B 2002, 106, 11474–11486. 10.1021/jp026217s.

[ref54] Knoester J. Modeling the optical properties of excitons in linear and tubular J-aggregates. Int. J. Photoenergy 2006, 2006, 1–10. 10.1155/IJP/2006/61364.

[ref55] Fujita T.; Brookes J. C.; Saikin S. K.; Aspuru-Guzik A. Memory-assisted exciton diffusion in the chlorosome light-harvesting antenna of green sulfur bacteria. J. Phys. Chem. Lett. 2012, 3, 2357–2361. 10.1021/jz3008326. 26292114

[ref56] Alvertis A. M.; Pandya R.; Muscarella L. A.; Sawhney N.; Nguyen M.; Ehrler B.; Rao A.; Friend R. H.; Chin A. W.; Monserrat B. Impact of exciton delocalization on exciton-vibration interactions in organic semiconductors. Phys. Rev. B 2020, 102, 081122 10.1103/PhysRevB.102.081122.

[ref57] Sardjan A. S.; Westerman F. P.; Ogilvie J. P.; Jansen T. L. C. Observation of ultrafast coherence transfer and degenerate states with polarization-controlled two-dimensional electronic spectroscopy. J. Phys. Chem. B 2020, 124, 9420–9427. 10.1021/acs.jpcb.0c08126. 32990439 PMC7586392

[ref58] Van Der Vegte C.; Dijkstra A.; Knoester J.; Jansen T. L. C. Calculating two-dimensional spectra with the mixed quantum-classical ehrenfest method. J. Phys. Chem. A 2013, 117, 5970–5980. 10.1021/jp311668r. 23360103

[ref59] Mukamel S. Principles of Nonlinear Optical Spectroscopy; Oxford University Press on Demand, 1999.

[ref60] Kramer T.; Rodríguez M. Effect of disorder and polarization sequences on two-dimensional spectra of light-harvesting complexes. Photosyn. Res. 2020, 144, 147–154. 10.1007/s11120-019-00699-6. 31872335

[ref61] Liang C.; Jansen T. L. C. An efficient N3-scaling propagation scheme for simulating two-dimensional infrared and visible spectra. J. Chem. Theory Comput. 2012, 8, 1706–1713. 10.1021/ct300045c. 26593664

[ref62] Hybl J. D.; Albrecht A. W.; Gallagher Faeder S. M.; Jonas D. M. Two-dimensional electronic spectroscopy. Chem. Phys. Lett. 1998, 297, 307–313. 10.1016/s0009-2614(98)01140-3.

[ref63] Vlaming S. M.; Bloemsma E. A.; Nietiadi M. L.; Knoester J. Disorder-induced exciton localization and violation of optical selection rules in supramolecular nanotubes. J. Chem. Phys. 2011, 134, 114507 10.1063/1.3528993. 21428632

[ref64] Bloemsma E.; Vlaming S.; Malyshev V.; Knoester J. Signature of Anomalous Exciton Localization in the Optical Response of Self-Assembled Organic Nanotubes. Phys. Rev. Lett. 2015, 114, 156804 10.1103/PhysRevLett.114.156804. 25933330

[ref65] Wan Y.; Stradomska A.; Fong S.; Guo Z.; Schaller R. D.; Wiederrecht G. P.; Knoester J.; Huang L. Exciton level structure and dynamics in tubular porphyrin aggregates. J. Phys. Chem. C 2014, 118, 24854–24865. 10.1021/jp507435a.

[ref66] Bondarenko A. S.; Jansen T. L. C.; Knoester J. Exciton localization in tubular molecular aggregates: Size effects and optical response. J. Chem. Phys. 2020, 152, 194302 10.1063/5.0008688. 33687267

[ref67] Kunsel T.; Günther L. M.; Köhler J.; Jansen T. L.; Knoester J. Probing size variations of molecular aggregates inside chlorosomes using single-object spectroscopy. J. Chem. Phys. 2021, 155, 124310 10.1063/5.0061529. 34598584

[ref68] Malina T.; Koehorst R.; Bína D.; Pšenčík J.; van Amerongen H. Superradiance of bacteriochlorophyll c aggregates in chlorosomes of green photosynthetic bacteria. Sci. Rep. 2021, 11, 8354 10.1038/s41598-021-87664-3. 33863954 PMC8052352

[ref69] Yang J.; Gelin M. F.; Chen L.; Šanda F.; Thyrhaug E.; Hauer J. Two-dimensional fluorescence excitation spectroscopy: A novel technique for monitoring excited-state photophysics of molecular species with high time and frequency resolution. J. Chem. Phys. 2023, 159, 074201 10.1063/5.0156297. 37581414

[ref70] Fidder H.; Knoester J.; Wiersma D. A. Optical properties of disordered molecular aggregates: A numerical study. J. Chem. Phys. 1991, 95, 7880–7890. 10.1063/1.461317.

[ref71] Molina R. A.; Benito-Matias E.; Somoza A. D.; Chen L.; Zhao Y. Superradiance at the localization-delocalization crossover in tubular chlorosomes. Phys. Rev. E 2016, 93, 022414 10.1103/PhysRevE.93.022414. 26986369

[ref72] Taffet E. J.; Fassioli F.; Toa Z. S.; Beljonne D.; Scholes G. D. Uncovering dark multichromophoric states in Peridinin–Chlorophyll–Protein. J. R. Soc. Interface 2020, 17, 20190736 10.1098/rsif.2019.0736. 32183641 PMC7115236

[ref73] Chuang C.; Bennett D. I.; Caram J. R.; Aspuru-Guzik A.; Bawendi M. G.; Cao J. Generalized Kasha’s model: T-dependent spectroscopy reveals short-range structures of 2d excitonic systems. Chem 2019, 5, 3135–3150. 10.1016/j.chempr.2019.08.013.

[ref74] Chuang C.; Cao J. Universal scalings in two-dimensional anisotropic dipolar excitonic systems. Phys. Rev. Lett. 2021, 127, 047402 10.1103/PhysRevLett.127.047402. 34355927

[ref75] Meneghin E.; Biscaglia F.; Volpato A.; Bolzonello L.; Pedron D.; Frezza E.; Ferrarini A.; Gobbo M.; Collini E. Biomimetic nanoarchitectures for light harvesting: Self-assembly of pyropheophorbide-peptide conjugates. J. Phys. Chem. Lett. 2020, 11, 7972–7980. 10.1021/acs.jpclett.0c02138. 32886518 PMC8011917

[ref76] Zhao H.; Mazumdar S.; Sheng C.-X.; Tong M.; Vardeny Z. Photophysics of excitons in quasi-one-dimensional organic semiconductors: Single-walled carbon nanotubes and π-conjugated polymers. Phys. Rev. B: Condens. Matter Mater. Phys. 2006, 73, 075403 10.1103/PhysRevB.73.075403.

[ref77] Efros A. L.; Rosen M.; Kuno M.; Nirmal M.; Norris D. J.; Bawendi M. Band-edge exciton in quantum dots of semiconductors with a degenerate valence band: Dark and bright exciton states. Phys. Rev. B: Condens. Matter Mater. Phys. 1996, 54, 4843–4856. 10.1103/PhysRevB.54.4843. 9986445

[ref78] Ranjbar Choubeh R.; Koehorst R. B.; Bína D.; Struik P. C.; Pšenčík J.; van Amerongen H. Efficiency of excitation energy trapping in the green photosynthetic bacterium Chlorobaculum tepidum. Biochim. Biophys. Acta Bioenerg. 2019, 1860, 147–154. 10.1016/j.bbabio.2018.12.004. 30537470

